# *Ex-vivo* Mechanical Testing of Novel Laryngeal Clamps Used for Laryngeal Advancement Constructs

**DOI:** 10.3389/fvets.2020.00139

**Published:** 2020-03-12

**Authors:** Remigiusz M. Grzeskowiak, James Schumacher, Pierre-Yves Mulon, Richard C. Steiner, Lynne Cassone, David E. Anderson

**Affiliations:** ^1^Department of Large Animal Clinical Sciences, College of Veterinary Medicine, The University of Tennessee, Knoxville, Knoxville, TN, United States; ^2^Veterinary Diagnostic Laboratory, College of Agriculture, Food and Environment, The University of Kentucky, Lexington, KY, United States

**Keywords:** equine, tie-forward, larynx, pharynx, laryngeal advancement, DDSP, upper airway, soft palate

## Abstract

Rostral laryngeal advancement, also known as laryngeal tie-forward, is used to treat horses for intermittent dorsal displacement of the soft palate and has a morbidity rate of about 6%. We hypothesized that a novel laryngeal clamp would prevent morbidity associated with the sutures tearing through the thyroid cartilage. Larynges (*n* = 35 horses) were used for *ex vivo* testing. For uniaxial testing, 15 equine larynges were tested in one of three laryngeal tie-forward constructs [standard laryngeal tie-forward; modified laryngeal tie-forward using a suture-button; and modified laryngeal tie-forward using a laryngeal clamp]. For biaxial testing, 20 larynges were tested in one of two treatment groups: laryngeal tie-forward and laryngeal tie-forward using a laryngeal clamp. Constructs were tested in single cycle-to-failure. Statistical analyses were performed using ANOVA for uniaxial testing and *t*-tests for biaxial testing. The laryngeal tie-forward using a laryngeal clamp construct was superior to laryngeal tie-forward and laryngeal tie-forward using a suture-button constructs in resistance to pullout in uniaxial testing. The laryngeal tie-forward using a laryngeal clamp presented a significantly different method of failure than the standard laryngeal tie-forward in the biaxial testing. Failure modes for each construct were primarily by suture failure at the clamp (laryngeal tie-forward using a laryngeal clamp), suture pullout through the thyroid cartilage, or, less commonly, tearing of the cricothyroid ligament (laryngeal tie-forward). In uniaxial testing, the laryngeal tie-forward using a laryngeal clamp failed most commonly due to tearing of the cricothyroid ligament, whereas the standard laryngeal tie-forward and the laryngeal tie-forward using a suture-button failed due to the tearing of the cartilage. The laryngeal clamps provided greater stiffness, load at yield, and tensile stress at yield than did the standard construct. Laryngeal clamps may offer an alternative to standard methods of anchoring the thyroid cartilage when performing the laryngeal tie-forward procedure. Further testing and clinical trials are needed to elucidate the utility of the laryngeal tie-forward using a laryngeal clamp.

## Introduction

Intermittent dorsal displacement of the soft palate (IDDSP) is a multifactorial disease occurring in 10–20% of racehorses (1–7) and 28% of competing draft horses (8). Females and young horses (2–4 years old) have been reported to be more affected than male and older horses (9). The etiology of this disease is multifactorial, and proposed causes include reduced activity of the thyrohyoideus muscles (10–12), reduced activity of the palatine and palatopharyngeal muscles (13), and reduced activity of the hypoglossal nerve (14).

The suggested medical and surgical treatments of affected horses are many, because the pathophysiology associated with IDDSP is complex (15–22). Surgical treatments include myectomy of the laryngeal retractor muscles (18–20), staphylectomy (21, 22), palatoplasty (21, 22), and rostral advancement of the larynx, commonly referred to as the laryngeal tie-forward (LTF) procedure (10–12). The success rate of the LTF procedure in preventing IDDSP is reported to be in the range of 80–82%, which is better than the rate associated with the other procedures (11, 12). This procedure advances the larynx dorsally and rostrally using a biaxial suture construct in which two non-absorbable sutures, one inserted through the caudoventral aspect of the right lamina of the thyroid cartilage and the other inserted in a similar manner through the left lamina of the thyroid cartilage and anchored to each other at the basihyoid bone, mimic the action of the thyrohyoideus muscles (10, 11). The incidence of complications associated with this surgery has been reported to be about 6%, and the primary complication has been the failure of one or both sutures to retain the larynx in its new position (23, 24).

The LTF procedure is most often performed using polyethylene or polyester sutures (10, 11, 25, 26), and according to previous reports, the procedure can fail if one or both sutures break or tear through the thyroid cartilage. The LTF procedure reported by Woodie et al. (11) and Cheetham et al. (12) has been modified recently in an attempt to increase the mechanical strength of the construct (5, 11, 26, 27). Rossignol et al. (27) modified the procedure by anchoring the suture to the thyroid cartilage with metallic implants (i.e., “suture buttons”; LTFB), which resulted in improvement of the mechanical stability of the construct during mechanical testing (27). Rossignol et al. (27) found, however, that the strength of the modified LTFB construct was inferior to that of the standard LTF construct and reported that the method of failure in both types of constructs was pullout of the suture from the thyroid cartilage (25). An implant that reduces the risk of a pullout from the thyroid cartilage is needed, because the method of failure of the LTF procedure in all tested constructs has been pullout of the construct through the thyroid cartilage.

We hypothesized that a custom-designed, novel laryngeal clamp (LTFC) would distribute tensile forces over a large area of the thyroid cartilage, thereby eliminating morbidity associated with failure at the suture-cartilage interface. The objectives of the study were to design and manufacture a novel laryngeal clamp and to study the mechanical characteristics of the LTF constructs (LTF, LTFB, LTFC) anchored to only one lamina of the thyroid cartilage (uniaxial testing) and to mechanically test the strength of two different constructs (LTF, LTFC) anchored to both laminae of the thyroid cartilage (biaxial testing). We expected that the difference in *ex vivo* uniaxial mechanical testing between the constructs would be significantly different, but testing between right-sided and left-sided constructs would not be significant. We expected the results of *ex vivo* mechanical testing between the standard construct and the construct modified with clamps would be significantly different in biaxial mechanical tests.

## Materials and Methods

### Preparation of the Larynxes

Thirty-five larynges, each with its hyoid apparatus attached, were collected from horses within 24 h after the horses were euthanized for a reason unrelated to the respiratory tract. Of these 35 larynges, 15 were used in proof-of-concept, uniaxial mechanical studies, and 20 were used in biaxial mechanical studies. Signalment data, including age, weight, breed, and sex, were available for horses supplying the larynxes (*n* = 20) used for biaxial testing. Larynges were frozen at −20°C until mechanical testing was performed (25, 27–32) and were thawed at room temperature for 24 h prior to testing. The larynges were wrapped in isotonic saline-soaked gauze after they were thawed to keep them moist until tested.

### Sample Measurements Collection

**Figure 1:**
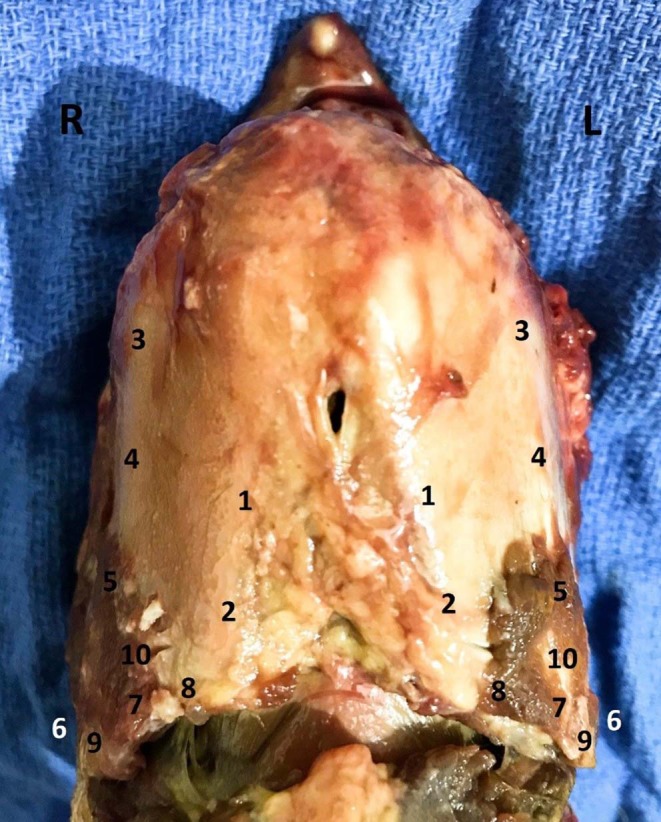
Thickness at 10 different anatomical positions. The thickest portion of the thyroid cartilage was found in positions 7–10, at the attachment of previously removed sternothyroideus muscle.

Before performing uniaxial tests, the thickness of the laminae of 15 thyroid cartilages was measured at 3 locations, 10 mm apart, on the caudoventral margin of the laminae, close to the insertion of the sternothyroideus muscles, using a digital caliper^1^. The thickness of the laminae of the 20 thyroid cartilages used for biaxial testing was measured in a similar fashion but in 10 different locations on each lamina to assess the properties of the thyroid cartilage (Figure 1, Table 1). Locations 1 and 2 were at the ventral and axial aspect of the thyroid cartilage. Locations 3 to 5 were at the dorsal aspect of the thyroid cartilage, and locations 6 to 10 were at the caudoventral aspect of the right and left laminae, close to the insertion of the sternothyroideus muscle. Measurements were obtained to determine the space between the arms of the U-shaped clamp necessary to insert the clamp onto the thyroid cartilage (Figure 1).

**Table 1:** Measurements of thickness in 10 anatomical positions.

**Thickness measurements (mm)**		
**Position**	**Mean**	**Std. Deviation**
1.00	3.8872	0.84690
2.00	3.9444	0.76652
3.00	3.9336	0.72256
4.00	3.5096	0.56269
5.00	3.7696	0.60627
6.00	3.9156	0.67555
7.00	6.0163	1.22410
8.00	4.9548	0.81034
9.00	4.4984	0.67038
10.00	6.4740	1.43194

### Uniaxial Testing

**Figure 2:**
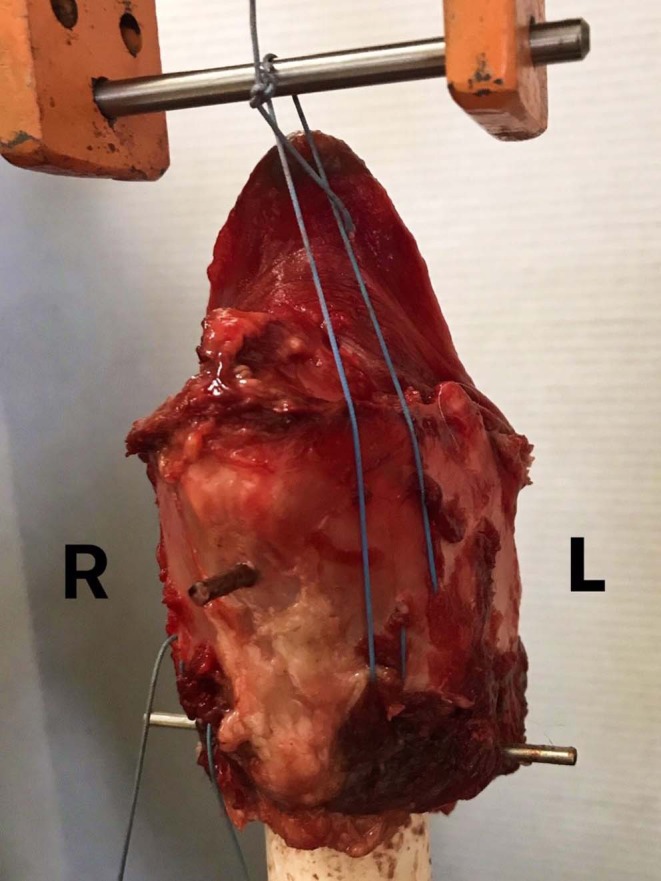
Single-side LTF mechanical testing. This figure presents the experimental arrangement of a larynx in the MTS testing machine. The larynx was attached to the 3.2 cm PVC cylinder, which was attached to the holding grip of the MTS machine. A custom-made holding grip with a stainless-steel bar was attached to the loading cell. Each larynx was in the same treatment group, but each side of the construct was tested separately. The ends of the suture were tightened and tied around the stainless-steel bar.

Fifteen larynges were randomly assigned to one of three treatment groups according to the construct tested, and each side of the larynx to which the construct was applied was tested separately. Uniaxial tests on the suture-cartilage interface included the following: (1) standard LTF using USP no. 5 polyester suture^2^, (2) LTF modified with a suture button (LTFB), and (3) LTF modified with a custom-designed laryngeal clamp (LTFC) (Figure 2). The uniaxial testing was performed to evaluate the performance of a single implant anchored to the thyroid cartilage and to assess the difference in the mechanical performance between the left and right laminae of the thyroid cartilage.

### Biaxial Testing

**Figure 3:**
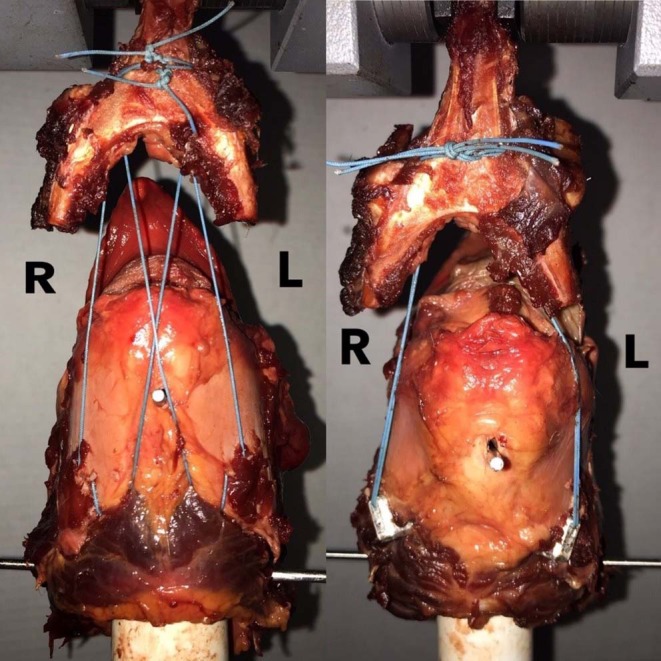
LTF vs. LTFC biaxial construct testing. This figure shows the arrangement of the LTF and LTFC constructs in the MTS machine. Larynges were mounted on a PVC cylinder, as in the single-side testing, and the basihyoid bone was attached to the holding grip of the loading cell. In the standard LTF construct, on the left side, the free ends of the ipsilateral dorsal suture and contralateral ventral suture were advanced rostrally and dorsally to the basihyoid bone. The same procedure was performed on the right side. The ventral and dorsal sutures were tightened and tied together around the ventral aspect of the lingual process of the basihyoid bone. In the LTF construct modified with the laryngeal clamps, both ends of the sutures were advanced rostrally and dorsal to the bone and tied together around the ventral aspect of the lingual process.

Twenty larynges were randomly assigned to one of two treatment groups according to the construct tested, and both sides of the larynx to which the construct was applied were tested simultaneously. The constructs tested included the LTF construct using USP no. 5 polyester suture^2^ and the LTFC. The ceratohyoid and thyrohyoid bones were removed from the basihyoid bone, which was mounted on the loading frame of an electromechanical testing machine^3^. The free ends of the sutures passing through the laminae of the thyroid cartilage were attached to the basihyoid bone (Figure 3).

### Laryngeal Tie-Forward (LTF)

The LTF procedure was performed in a manner similar to the technique described by Woodie et al. (11) and Cheetham et al. (12). Using this technique, USP No. 5 polyester suture^2^ was inserted twice through the right or left lamina of the thyroid cartilage, at the caudoventral aspect of the lamina, using a ½-circle, trocar-point, needle^4^ to create a single loop around the caudoventral margin of the lamina close to the tendon of insertion of the sternothyroideus muscle (Figure 3).

### Laryngeal Tie-Forward Modified With Suture Buttons (LTFB)

**Figure 4:**
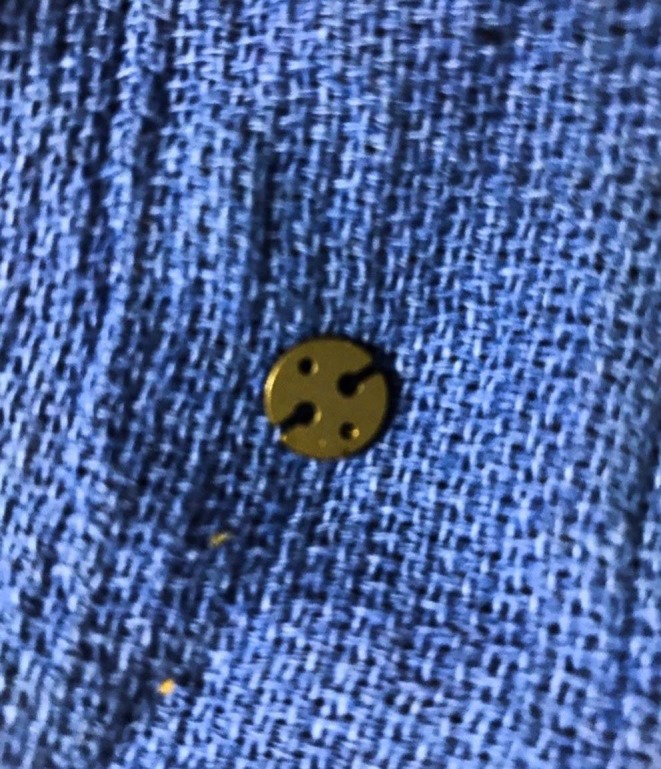
Suture Button. The commercially available suture button used for this study was a part of the kit for the human cruciate ligament repair. The surgical technique used to anchor the thyroid cartilage was performed as described by Rossignol et al. (27).

The LTFB procedure was performed in a manner similar to the technique described by Rossignol et al. (27). Using this technique, USP No. 5 polyester suture^2^ was threaded through the eyelet of the suture-button (2 mm thick rounded plate with 2, 1.5 mm diameter holes), and the free ends of the sutures were inserted through the eye of a ½-circle, trocar-point needle^4^. The needle was passed once through the caudoventral margin of the right or left lamina of the thyroid cartilage in a manner similar to that described using the LTF, and by applying tension to the suture, the button became seated firmly on the ventral aspect of the caudal edge of the thyroid cartilage (Figure 4).

### Laryngeal Tie-Forward Modified With Novel Laryngeal Clamp (LTFC)

**Figure 5:**
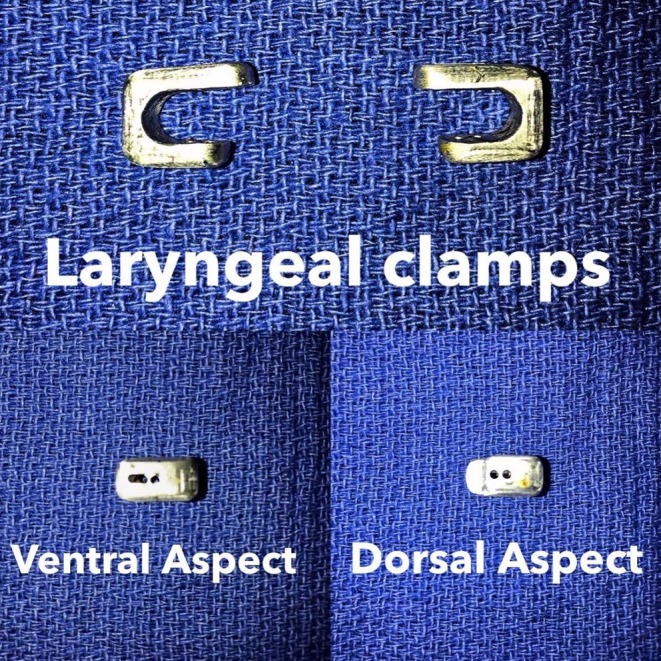
The lateral, dorsal, and ventral view of the laryngeal clamps. The design of laryngeal clamps aimed at creating an anchor that wrapped around the caudal border of the thyroid cartilage to distribute the tensile forces applied to the suture. The laryngeal clamps were U-shaped, 5 mm wide, and 1 cm long. The short arm was placed on the dorsal surface of the thyroid cartilage, and the long arm was placed on the ventral surface of the cartilage. The part connecting the bottom and top parts was responsible for distributing the tensile force. The short, dorsal arm had two holes, creating an eyelet for the suture, and the longer ventral arm had a slit, though which suture could easily be passed.

**Figure 6:**
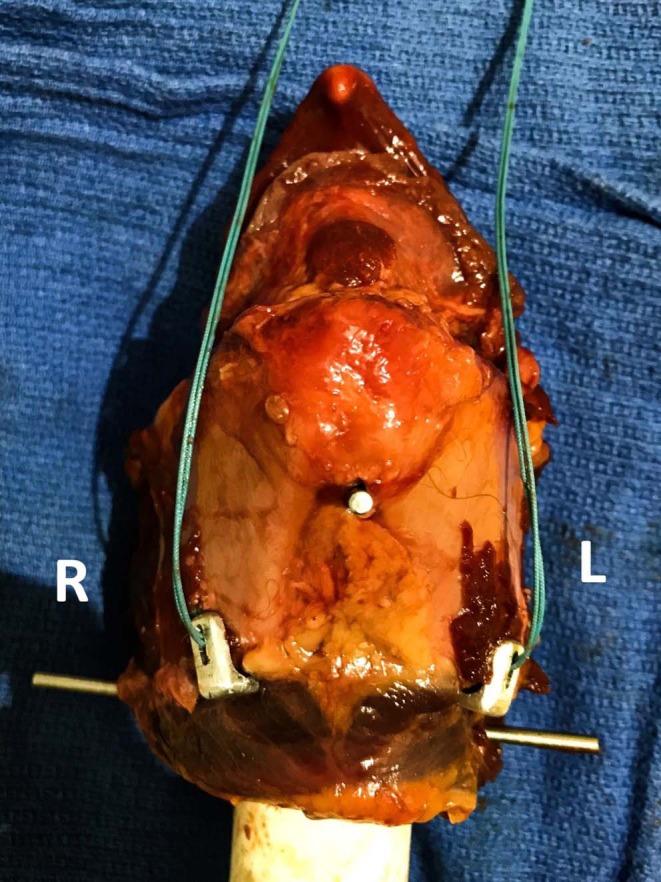
Laryngeal clamps positioned on the caudoventral margin of the thyroid cartilage. The figure shows the arrangement of the laryngeal clamp around the caudoventral border of the thyroid cartilage. The dorsal arm had an eyelet with the relief between two holes, through which the suture was threaded before the clamp was placed. The ends of the suture were threaded through the eye of a ½-circle needle, which was passed through the caudal margin of the thyroid cartilage. After the needle was passed, the ends of the suture were threaded through the slit on the dorsal arm of the clamp. Tension applied to the suture caused the clamp to engage the caudal margin of the lamina of the thyroid cartilage. The suture of this construct had no dorsal and ventral arms, because both arms were inserted through the one hole in the lamina of the thyroid cartilage.

The laryngeal clamps, composed of 316-stainless steel, were a broad U-shaped device designed to wrap around the caudoventral border of the laminae of the thyroid cartilage. One arm of the clamp was 10 mm long, the other was 14 mm long, and the gap with the “U” between the arms was 7 mm (Figure 5). The clamp was inserted around the caudoventral border of the right or left lamina of the thyroid cartilage close to the site of insertion of the sternothyroideus muscle, so the short arm lay dorsal to the lamina and the long arm ventral to the lamina. The dorsal arm contained two, 2-mm diameter holes spaced 2 mm apart, which together served as an eyelet for the suture, and the ventral arm contained a slit through which the suture was easily passed (Figure 5). The suture was attached to the clamp by threading one end of the suture through one hole in the eyelet and the other end through the adjacent hole, so the ends emerged between the arms of the clamp. The ends of the suture were then threaded through the eye of a ½-circle, trocar-point needle^4^, which was passed once through the right or left lamina of the thyroid cartilage close to the insertion of the sternothyroideus muscle. The needle emerged ~5–10 mm rostral to the caudal margin of the lamina (Figure 6). The needle was removed, and the ends of the suture were passed through the slit in the dorsal arm of the clamp. The clamp became seated firmly around the caudal edge of the thyroid cartilage at the site of insertion of the sternothyroideus muscle when tension was applied to the suture (Figure 6).

### Uniaxial Biomechanical Testing

Laryngeal constructs were tested in a single cycle-to-failure test using an electromechanical testing system with a 5-kN maximum load cell^3^. The larynges were mounted to the machine as previously described (25). Briefly, a 3.2-cm diameter cylinder of polyvinyl chloride (PVC) was passed into the lumen of the larynx, and two 3.5 mm diameter, 40 cm long Steinmann pins were placed through the larynx and cylinder perpendicular to the long axis of the cylinder and to each other. One pin was placed through the cricoid cartilage, and a second pin through the center of the thyroid cartilage, securing both cartilages firmly to the PVC cylinder, avoiding interference with the cricothyroid ligament.

After mounting the cylinder and larynx on the loading frame of the electromechanical testing machine with the rostral portion of the larynx uppermost (Figure 2), the ends of the suture were tied together around a 5 mm diameter steel bar attached to a custom-made holding grip, with a surgeon's knot, and secured with 7 throws (Figure 2). The suture loop resided in the center of the steel bar during the testing of each specimen, to mimic the direction of the suture *in vivo* and to maintain the repeatability of the testing conditions between the specimens. The pullout tests were performed at a distraction rate of 300 mm/min (25). The variables recorded for each construct included maximum load (N), stiffness (N/mm), extension at maximum load (mm), extension at the break (mm), extension at yield (mm), load at yield (N), tensile stress at yield (N/mm^2^), and tensile stress at maximum load (N/mm^2^). The maximum load was defined as the maximum force loaded to the point of failure of any part of the construct, and the stiffness was calculated from a load-displacement curve. Displacement was defined as a change in distance between the thyroid cartilage and steel bar, and the tensile stress was defined as the force applied to the area of the thyroid cartilage.

Three different modes of construct failure were observed and recorded, including tearing of the thyroid cartilage, tearing of the cricothyroid ligament, and breaking of the suture. The thyroid cartilage tore when the implant pulled through the cartilage. The cricothyroid ligament tore when the implant caused the thyroid cartilage to elevate and separate from the cricoid cartilage by tearing the cricothyroid ligament, resulting in little or no damage to the thyroid cartilage. Breaking of the suture, the third mode of failure, occurred at the knot of the loop around the steel bar.

### Biaxial Biomechanical Testing

The constructs tested in biaxial loading mimicked one of two clinical constructs, LTF and LTFC. Both constructs were anchored and mounted on the Instron machine^3^ in the manner described above. After removing the hyoid apparatus, the larynx was mounted on the upper loading cell of the electromechanical testing machine. For the LTF construct, the free end of the ipsilateral dorsal arm of the suture and contralateral ventral arm of the suture inserted through one lamina of the thyroid cartilage were advanced rostrally and dorsally to the detached basihyoid bone, which was mounted on the loading frame of the MTS testing machine^3^. The same procedure was performed on the contralateral side. The ventral arms of the right and left sutures were tied to each other around the ventral aspect of the lingual process, with a surgeon's knot, and secured with 7 throws. The dorsal arms of the right and left sutures were tied together in a similar fashion (Figure 3).

To create the LTFC construct, both ends of each suture were advanced rostrally and dorsally to the ipsilateral side of the basihyoid bone (Figure 3). The four arms of the sutures were tied together around the ventral aspect of the lingual process of the basihyoid bone, with a surgeon's knot, and secured with 7 throws. The pullout test was performed in a manner similar to that performed for uniaxial testing at a distraction rate of 300 mm/min. Three different modes of construct failure were observed and recorded and were the same as those observed during uniaxial testing.

### Statistical Analyses

Statistical analyses were performed by using IBM SPSS Statistics 25^5^. Descriptive statistics were calculated for all variables for all treatment groups and included minimum, maximum, mean, and standard deviation. The normality of data distribution for each variable was performed by using the Kolmogorov-Smirnov test of normality.

The thickness of the 3 sites measured on laminae of the thyroid cartilage of the larynges used for uniaxial testing was compared by using a two-tailed independent samples *T*-test. The thickness of the 10 sites measured on the right lamina of the thyroid cartilage of the larynges used for biaxial testing was compared to the thickness of the left lamina by using a two-tailed independent samples *T*-test. The results were examined for homogeneity of variance, and each site was compared by using ANOVA and the Tukey's *post-hoc* test. The thickness was also correlated with the age, breed, and weight of donors by using a two-tailed Pearson correlation.

For uniaxial tests, the differences in maximum load, stiffness, extension at maximum load, extension at break, extension at yield, load at yield, tensile stress at yield, and tensile stress at maximum load between the left and right testing side of the thyroid cartilage were examined for homogeneity of variance. The differences between each testing group were then analyzed by using ANOVA and Tukey's *post-hoc* test. The correlation between the construct and method of failure was examined by using Fisher's exact tests.

For biaxial tests, the differences between two constructs were compared by using a two-tailed independent samples *T*-test, and the correlation between the construct and method of failure was tested by using the Fisher's exact test. The level of statistical significance was established at *p* < 0.05 and the power of the experiment design and test combination was calculated using PS software^6^ in the reference to the results of maximum load during mechanical testing.

## Results

### Study Population

The weight of the horses from which the 35 larynges were harvested ranged from 450 to 650 kg (mean 570 ± 70.7 kg), and the ages of these horses ranged from 2 to 29 years old (mean 14.8 ± 8.1 years). Information about the breed and sex of the donors was obtained only for the 20 larynges used in biaxial tests. The breeds of these 20 horses included Thoroughbred (11), American Quarter Horse (3), Tennessee Walking Horse (1), Warmblood (2), American Standardbred (1), Hanoverian (1), and Clydesdale (1). Ten donors were female, and 10 were geldings.

### Sample Measurements Collection

The Kolmogorov-Smirnov test of normality of distribution showed the measurements of cartilage thickness to be normally distributed in all tested positions (*p* > 0.05). The Levene's test for equality of variances revealed that equal variances in all variables could be assumed (*p* > 0.05). The descriptive statistics of the thickness measurements of the thyroid cartilage of the 15 larynges used in the single-side testing (i.e., uniaxial tests) revealed that the mean thickness of the caudoventral edge of the left thyroid cartilage lamina was 4.58 ± 0.7 mm. The mean thickness of the caudoventral edge of the right thyroid cartilage lamina was 4.70 ± 0.7 mm (**Table 3**). The measurements of the caudoventral edge of the thyroid cartilage of the 10 larynges used in biaxial testing revealed that the mean thickness of the caudoventral edge of the left thyroid cartilage lamina was 4.48 ± 1.3 mm. The mean thickness of the caudoventral edge of the right thyroid cartilage lamina was 4.50 ± 1.2 mm. The differences in the average thickness between the left and right laminae of the thyroid cartilage in the study population were not statistically significant (*p* > 0.05) (Tables 2, 3).

**Table 2:** Comparison of thickness between the left and right laminae of the thyroid cartilage.

**2-tailed independent samples** ***T*****-test between left and right thyroid cartilage**				
	**Side**	**Mean**	**Std. Deviation**	**Sig. (2-tailed)**
Position_1	L	3.8872	0.84690	0.278
	R	4.1456	0.81930	0.278
Position_2	L	3.9444	0.76652	0.372
	R	3.7652	0.63383	0.372
Position_3	L	3.9336	0.72256	0.563
	R	4.0536	0.73439	0.563
Position_4	L	3.5096	0.56269	0.332
	R	3.6696	0.59214	0.332
Position_5	L	3.7696	0.60627	0.910
	R	3.7496	0.64440	0.910
Position_6	L	3.9156	0.67555	0.791
	R	3.9692	0.74584	0.791
Position_7	L	6.0163	1.22410	0.716
	R	5.8872	1.23921	0.716
Position_8	L	4.9548	0.81034	0.856
	R	4.9972	0.82942	0.856
Position_9	L	4.4984	0.67038	0.535
	R	4.6244	0.75340	0.535
Position_10	L	6.4740	1.43194	0.495
	R	6.1808	1.57979	0.495

*There were no significant differences between the left and right laminae at any of the measured positions*.

**Table 3:** Comparison of biomechanical results obtained during uniaxial testing involving the left or right laminae of the thyroid cartilage (2-tailed independent samples *T*-test).

**2-tailed independent samples** ***T*****-test between left and right thyroid cartilage**				
	**Side**	**Mean**	**Std. Deviation**	**Sig. (2-tailed)**
Average Thickness	L	4.5833	0.73626	0.670
	R	4.6987	0.73201	
Maximum Load (N)	L	190.4900	34.96656	0.614
	R	197.6793	41.82675	
Stiffness (N/mm)	L	4.0580	1.40277	0.884
	R	4.1240	1.03332	
Extension at Failure (mm)	L	56.1173	17.44167	0.627
	R	52.9907	17.37226	
Extension at Max Load (mm)	L	55.7880	17.60501	0.461
	R	51.1160	16.65160	
Extension at Yield (mm)	L	38.8507	14.03225	0.637
	R	41.1693	12.53842	
Load at Yield (N)	L	151.7260	45.32798	0.604
	R	159.9007	39.84170	
Tensile Stress at Yield (N/mm^2^)	L	15.1747	4.53286	0.605
	R	15.9900	3.98632	
Tensile Stress at Max Load (N/mm^2^)	L	18.8954	3.20007	0.535
	R	19.7723	3.86754	

*The data obtained from the left side of the larynges did not differ significantly from that obtained from the right side of the larynges*.

Significant differences were found in the thickness of the laminae of the thyroid cartilage among the different anatomical positions (*p* < 0.05). The position numbers 7 (L−6.01 ± 1.2 mm; R−5.89 ± 1.2 mm), 8 (L−4.95 ± 0.8 mm; R−4.99 ± 0.8 mm), and 10 (L−6.47 ± 1.4 mm; R−6.18 ± 1.6 mm) were significantly thicker than the rest of the anatomical positions (*p* < 0.05) (Table 1). These sites were at the caudal margin of the thyroid cartilage at the site of insertion of each sternothyroideus muscle (Figure 1). No significant correlation was found between the mean thickness of the caudoventral aspect of the margin of the laminae of the thyroid cartilage and the age and breed of the donors for any sites examined (*p* > 0.05). There was, however, a significant positive correlation between the mean thickness of the laminae and the weight of the donors (*r* = 0.78, *p* < 0.05) (Supplementary Table 1).

### Uniaxial Biomechanical Testing

Data was normally distributed and homogeneous for all variables (*p* > 0.05). The two-tailed independent samples *T*-test did not reveal significant differences in the results of biomechanical testing between the left and right side of the lamina (*p* > 0.05) (Table 3). The yield on the load-displacement curve corresponded to the tearing of the thyroid cartilage (*n* = 22) or to the tearing of the cricothyroid ligament (*n* = 7).

**GRAPH 1:**
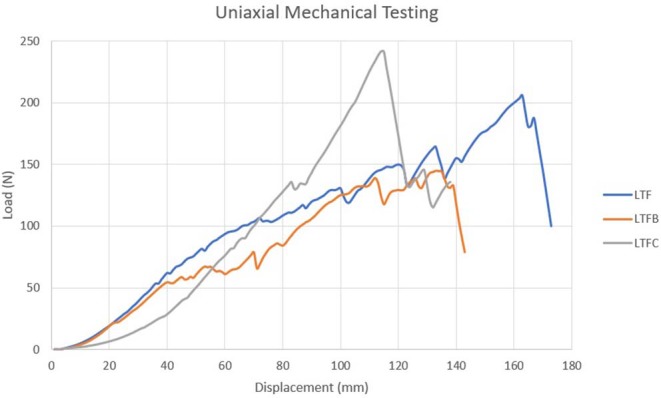
The graph compares the representative biomechanical performance of the uniaxial constructs anchored to the left lamina of the thyroid cartilage. The uniaxial LTFC construct (gray line) attained the highest maximum load followed first by the LTF and then LTFB construct. The method of failure associated with LTFC construct was tearing of the cricothyroid ligament, whereas the LTF and LTFB constructs failed because the thyroid cartilage tore. The LTFC construct protected the cartilage.

Uniaxial testing of the constructs revealed that the mean pullout strengths of the LTFC (207 ± 43 N) and LTF (199 ± 19 N) constructs were significantly greater than the mean pullout strength of the LTFB construct (176 ± 43 N) (*p* < 0.05) and that the difference in the mean pullout strength of the LTFC construct did not differ significantly from that of the LTF constructs (*p* > 0.05). Similarly, the mean load at yield point was significantly greater for the LTFC (161 ± 45 N) and LTF (168 ± 23 N) constructs than that for the LTFB construct (139 ± 51 N) (*p* < 0.05), but the mean load at yield point for the LTFC construct did not differ significantly from that the of the LTF construct. The results of the rest of the uniaxial mechanical testing were not significantly different between the treatment groups (*p* > 0.05) (Table 4, Graph 1).

**Table 4:** The comparison of the results of biomechanical testing between three constructs in the single-side testing.

**ANOVA of three different constructs**				
	**Construct**	**Mean**	**Std. Deviation**	**Sig**.
Maximum Load (N)	LTF	199.0180	19.27660	0.024
	LTFB	176.3040	43.18806	
	LTFC	206.9320	43.21904	
Stiffness (N/mm)	LTF	3.8600	0.93406	0.177
	LTFB	4.7650	1.36203	
	LTFC	3.6480	1.10265	
Extension at Failure (mm)	LTF	55.6220	12.66911	0.087
	LTFB	46.2630	14.58875	
	LTFC	61.7770	21.02075	
Extension at Max Load (mm)	LTF	53.0860	11.89288	0.125
	LTFB	45.9100	14.71806	
	LTFC	61.3600	20.99364	
Extension at Yield (mm)	LTF	46.0220	12.83827	0.125
	LTFB	29.2360	8.72437	
	LTFC	44.7720	10.95712	
Load at Yield (N)	LTF	167.5140	23.34335	0.003
	LTFB	138.5570	51.10873	
	LTFC	161.3690	45.24054	
Tensile Stress at Yield (N/mm^2^)	LTF	16.7520	2.33487	0.278
	LTFB	13.8570	5.11180	
	LTFC	16.1380	4.52568	
Tensile Stress at Max Load (N/mm^2^)	LTF	19.8430	1.86375	0.279
	LTFB	18.1510	4.24579	
	LTFC	20.4567	4.23621	

*The results were analyzed by using ANOVA. This table shows the mean values and standard deviations of the results of biomechanical testing of 3 constructs. The results differed significantly in maximum load (N) and load at yield (N) between the LTFC, LTF and LTFB constructs. The LTFC and LTF constructs had higher maximum load and load at yield than did the LTFB constructs, however the results did not differ significantly between the LTFC and LTF constructs*.

Fisher's exact test revealed a significant correlation between the construct and method of failure (*p* < 0.05). Of the constructs modified with a laryngeal clamp, 70% failed by tearing the cricothyroid ligament, and 30% failed by tearing the thyroid cartilage adjacent to the clamp. None of these constructs failed from the break of the suture. All (100%) of the LTF constructs failed from tearing the thyroid cartilage. Constructs modified with a suture button failed from tearing the thyroid cartilage (90%) or from breaking of the suture (10%) (Supplementary Table 2).

### Biaxial Biomechanical Testing

All variables, including biomechanical values, were normally distributed (*p* > 0.05). Tests for equality of variances revealed that equal variances in all variables could be assumed (*p* > 0.05).

**GRAPH 2:**
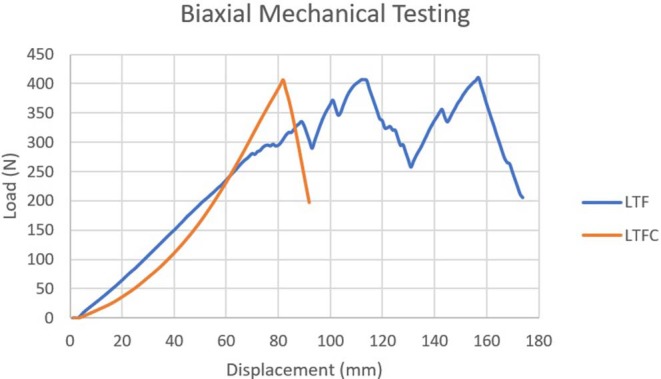
The graph compares the representative biomechanical performance of the biaxial LTF construct (blue line) to that of the biaxial LTFC construct (orange line). The yield point of the LTF construct corresponded to the moment at which the construct started to tear the thyroid cartilage. The first peak in this graph represents tearing of the thyroid cartilage, and the second peak represents tearing of the cricothyroid ligament. The LTFC construct failed when the suture failed its attachment to the laryngeal clamp; no damage to thyroid cartilage was noticed at the point of failure. The laryngeal clamp provided higher stiffness, force, and tensile stress at yield point and a different method of failure, which was significantly associated with the suture breaking.

As occurred in the uniaxial testing, the yield on the load-displacement curve corresponded to tearing of the thyroid cartilage (*n* = 8) or to tearing of the cricothyroid ligament (*n* = 3). The LTF construct compared to the LTFC in the biaxial testing had a significantly higher mean maximum load (430.3 ± 64.84 and 367.9 ± 55 N, respectively), lower mean extension at yield (33.38 ± 8.9 and 42.54 ± 7.81 mm, respectively), higher mean extension at maximum load (64.9 ± 9.5 and 52.4 ± 8.4 mm, respectively), higher mean extension at failure (65.4 ± 9.5 and 52.6 ± 8.5 mm, respectively), higher mean extension at maximum load (64.9 ± 9.5 and 52.4 ±8.4 mm, respectively) and higher mean tensile stress at maximum load (43.13 ± 6.87 and 36.8 ± 9.1, N/mm^2^) (*p* < 0.05) (Table 5). The results of the rest of the biaxial mechanical testing were not significantly different between the treatment groups (*p* > 0.05) (Table 5, Graph 2).

**Table 5:** The comparison between the performance of two biomechanically tested constructs using a 2-tailed independent samples *T*-test.

**2-tailed independent samples T-test between two constructs**				
	**Construct**	**Mean**	**Std. Deviation**	**Sig. (2-tailed)**
Average Thickness (mm)	LTF	4.6010	0.33834	0.500
	LTFC	4.7880	0.79014	
Maximum Load (N)	LTF	430.2940	64.84309	0.032
	LTFC	367.8790	55.19138	
Extension at Failure (mm)	LTF	65.3910	9.47112	0.005
	LTFC	52.5820	8.49355	
Extension at Max Load (mm)	LTF	64.8970	9.52261	0.006
	LTFC	52.4210	8.44992	
Extension at Yield (mm)	LTF	33.3780	8.98399	0.026
	LTFC	42.5410	7.81333	
Load at Yield (N)	LTF	264.6490	65.42587	0.257
	LTFC	306.2130	91.09641	
Tensile Stress at Max Load (N/mm^2^)	LTF	43.1300	6.86934	0.040
	LTFC	36.8210	9.10953	
Tensile Stress at Yield (N/mm^2^)	LTF	26.4660	6.54315	0.257
	LTFC	30.6210	5.51279	
Stiffness (N/mm)	LTF	6.7250	1.12806	0.640
	LTFC	7.0420	1.78153	

*The LTFC construct was inferior to the LTF construct in maximum load (p < 0.05), extension at failure (p < 0.05), extension at maximum load (p < 0.05), and tensile stress at maximum load (p < 0.05). The LTFC construct showed, however, a superior trend compared to the LTF construct in extension at yield (p < 0.05). The difference between the constructs in the remaining results were not significant (p > 0.05)*.

**Figure 7:**
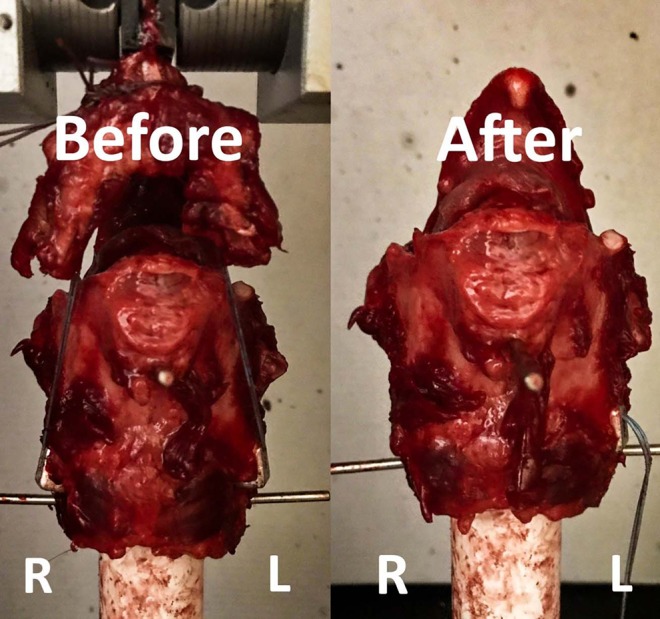
This figure shows testing of the LTFC and LTF constructs in the Instron machine. The image on the left shows the construct before the testing, and the image on the right shows the construct after the testing. The left and right laminae of the thyroid cartilage are labeled. Notice that the appearance of the thyroid cartilage on the right looks similar the appearance of the thyroid cartilage before the test. In this case, the right laryngeal clamp pulled out from the thyroid cartilage without inducing severe disruption of the crico-thyroid ligament and alteration of the geometry of the thyroid cartilage.

There was a significant correlation between the construct and method of failure (*p* < 0.05). Of constructs modified with clamps, 80% failed because the suture broke, and 20% failed because the cricothyroid ligament tore (Figure 7). Standard LTF constructs failed because the thyroid cartilage tore (80%) or because the cricothyroid ligament tore (20%) (Supplementary Table 2).

## Discussion

The mode of failure of the construct modified with laryngeal clamps differed from the mode of failure of the LTF construct. The mode of failure of the constructs correlated with the type of implant used, regardless of whether one side of the larynx or both sides of the larynx were tested. The LTF construct and the LTFB construct failed in uniaxial testing most commonly by pulling of the implant through the thyroid cartilage. Less commonly, the construct failed when the cricothyroid ligament tore. The LTF construct failed in biaxial testing when the suture pulled through the thyroid cartilage. The construct with the laryngeal clamps failed in uniaxial testing when the thyroid cartilage elevated and separated from the cricoid cartilage at the cricothyroid ligament. All constructs with the laryngeal clamps failed in biaxial testing when the suture broke close to one of the laryngeal clamps. The laryngeal clamps, therefore, protected the thyroid cartilage from tearing during uniaxial and biaxial testing.

The mode of failure of the biaxial construct modified with clamps may have perhaps been different, and the pullout force higher, if polyethylene suture, rather than polyester suture, had been used. Polyethylene suture has been shown to provide greater resistance to fraying, higher tensile strength, and more elasticity than the polyester suture used in both trials in our study (26, 33). Although polyethylene sutures performed better than polyester sutures in another similar study, the difference in the performance of the entire construct was not statistically significant, because the failure of the construct was due to failure of the thyroid cartilage, rather than a failure of the suture (25). A study examining the mechanical properties of various suture materials found polyethylene sutures to have a 500-fold greater resistance to fraying than polyester sutures (33). This makes polyethylene sutures particularly advantageous for attaching to metallic anchors or to absorbable anchor eyelets (32). Comparing the mechanical performance of various types of sutures used with the laryngeal clamp was beyond the scope of this study.

The force required to disrupt the LTFC constructs was similar to that of the LTF and LTFB constructs in uniaxial mechanical testing. The differences in the properties of biological samples, including a difference in viscoelasticity, may explain the high variability of force required to disrupt the constructs within each group. In biaxial construct testing, however, the pullout force for the LTF constructs was greater than the pullout force for the LTFC constructs. The LTFB construct was not included in biaxial testing because it performed more poorly in the single-side testing than the LTF construct. In a recent biomechanical study, LTF constructs had a higher pullout force in biaxial testing than did constructs modified with suture buttons, regardless of whether the suture was polyethylene or polyester (25).

The types of constructs failed at different forces and displacement at yield, because of the different modes at which they failed. The yield point in the mechanical testing corresponded with the force and displacement at which the cartilage or the cricothyroid ligament started to fail. Interestingly, the extension at which the construct failed differed distinctly among the types of constructs. The force and displacement at yield was highest for the LTF construct in the uniaxial testing followed next by the LTFC construct and then by the LTFB construct. The load at yield was higher for the LTFC construct than for the LTF construct in the biaxial testing. The higher load at yield resulted in a greater displacement at yield for the LTFC construct than for the LTF construct but not significantly so. The yield point of the LTFC constructs was rarely observed, because the entire construct moved in the Instron machine until the suture failed, whereas the yield point of the LTF construct was easily observed, because the suture, rather than the entire construct, moved until the yield point of the construct was reached, which was that point at which the thyroid cartilage began to tear.

The lower tensile force required to tear the cartilage in the LTF construct was related to the higher shear stress applied directly to the thyroid cartilage by the suture. The laryngeal clamp reduced stress by distributing the tensile force over a larger area of the thyroid cartilage. Yield points have not been reported in previous mechanical studies examining LTF constructs (25–27). The study performed by Santos et al. (26) examined the force at 20 and 30 mm extensions (25). We found the yield point to be about 34 mm for the LTF construct and about 42 mm for the LTFC construct, but the wide standard deviation for the LTFC construct suggests that the yield point was not constant for all tested constructs.

The stiffness of the LTFB construct was higher than that of the LTF and LTFC constructs in the single-side testing, causing the LTFB to construct to have the shortest extension when the suture broke. The LTF and LTFC constructs had a similar extension at failure in the uniaxial testing. The extension at failure for the LTFB construct, however, was higher than that reported by Santos et al. (25), though the trend in their study was similar. The LTFC constructs were stiffer than the LTF constructs in biaxial testing, resulting in a lower extension when the suture broke. The results of our tests showed that the LTFC constructs were stiffer than the LTF constructs in the single-side testing and the biaxial testing, and therefore, more force was required to disrupt the construct modified with clamps. The higher stiffness of the LTF constructs modified with the laryngeal clamps is likely to be associated with less laryngeal displacement under *in vivo* conditions.

Clinical failure of the LTF construct is reported to occur most commonly during recovery from general anesthesia when the horse rapidly and forcefully extends its head (25). In our study, the constructs modified with the laryngeal clamps had a pullout force of approximately 5.5 times that estimated to occur during maximal head extension (25). The tensile stress at maximum load was significantly lower for the LTFC construct than for the LTF construct, but the tensile stress at yield was higher for the LTFC constructs than for the LTF constructs. This may suggest that the tensile strength of the LTFC construct was greater at the moment when the cartilage or cricothyroid ligament began to fail. In the majority of the LTFC constructs, the suture, rather than the cartilage or the ligament, failed, resulting in a short yield phase just before failure. The tensile stress applied to the constructs under *in vivo* conditions is not known, and therefore, the minimal stress the constructs must withstand is not known.

The focus of this study was to evaluate the biomechanical performance of the LTF construct modified with a novel laryngeal clamp and to compare it to currently used constructs. One of the concerns with the novel design of the laryngeal clamps is the tension applied to the sutures, which results in shear stress between the sutures and metallic clamps during cyclical loading. The design tried to address these concerns and apply modifications aimed at reducing the shear stress between the suture and implant. Future research should examine additional modifications to reduce the shear stress on the suture to improve the performance of the LTFC construct. Also, the use of different sutures, such as polyethylene sutures, to anchor the clamps should be examined. The limitation of this study was the underpowered (β <0.8) design and test combination to detect hypothetical differences in mechanical properties between the constructs. Accounting for the difference in the mean values of maximum loads between the constructs and the standard deviations within the treatment groups, the study would necessitate 237 samples per treatment group to achieve study power β> 0.8. Furthermore, this is an *ex vivo* study in which each of the larynges had to undergo one freeze-thaw cycle which could have influenced the mechanical performance of the thyroid cartilage or the cricothyroid ligament. The results of this study might differ from the physiologic conditions and should be therefore interpreted with caution.

The prototype of the laryngeal clamp may offer an alternative to suture anchoring in the thyroid cartilage in LTF constructs. The unique design of the laryngeal clamp protected the cartilage by reducing the shear stress of the suture applied to the cartilage, which resulted in the higher force and tensile strength at the failure of the cartilage or ligament. This was associated with the increase in stiffness of the construct. Increasing the stiffness of the construct could result in the lower displacement of the construct over time *in vivo*. The increased stiffness of the construct modified with the clamps resulted in the sutures failing at lower maximum force and displacement.

## Data Availability Statement

All datasets generated for this study are included in the article/Supplementary Material.

## Ethics Statement

The study was conducted on the specimens obtained from the animals which were euthanized for reasons unrelated to the study. Therefore an ethical review process was not required for this study.

## Author Contributions

RG organized and analyzed data, prepared the specimens, and performed the laryngeal clamps placement procedures and the biomechanical testing as well as wrote the manuscript. JS performed the laryngeal clamps placement procedures, assisted with the biomechanical testing, and mentored the project. P-YM and RS created the method of biomechanical testing as well as performed the tests and helped with data collection and analysis. LC organized and prepared the specimens for testing. DA mentored the project as well as supervised data collection, analysis, and manuscript writing.

### Conflict of Interest

Pending patent for the Laryngeal Clamps. The authors declare that the research was conducted in the absence of any commercial or financial relationships that could be construed as a potential conflict of interest.

## Footnotes

^1^Husky Digital Caliper, The Home Depot, Atlanta, GA.

^2^Covidien, 710 Medtronic Parkway, Minneapolis, MN.

^3^Instron 5900, Instron, Norwood, MA.

^4^Richard-Allan, cat. no. 217001, Aspen Surgical, Caledonia, MI.

^5^SPSS ver. 25, IBM, 1 New Orchard Road Armonk, NY.

^6^PS Power and Sample Size Calculations ver 3.0, Vanderbilt University, Department of Biostatistics.

## Abbreviations

IDDSP: Intermittent Displacement of the Soft Palate
LTF: Laryngeal Tie-forward
LTFB: Laryngeal Tie-forward modified with suture buttons
LTFC: Laryngeal Tie-forward modified with laryngeal clamps
PVC: Polyvinyl Chloride.

## References

[1] Parente EJ Martin BB Tulleners EP Ross MW. Dorsal displacement of the soft palate in 92 horses during high-speed treadmill examination (1993-1998). Vet Surg. (2002) 31:507–12. 10.1053/jvet.2002.36009 12415518

[2] Dart AJ Dowling BA Hodgson DR Rose RJ Evaluation of high-speed treadmill video endoscopy for diagnosis of upper respiratory tract dysfunction in horses. Aust Vet J. (2001) 79:109–12. 10.1111/j.1751-0813.2001.tb10713.x 11256279

[3] Tan RH Dowling BA Dart AJ High-speed treadmill video endoscopic examination of the upper respiratory tract in the horse: the results of 291 clinical cases. Vet J. (2005) 170:243–8. 10.1016/j.tvjl.2004.06.011 16129344

[4] Lane JG Bladon B Little DR Naylor JR Franklin SH. Dynamic obstructions of the equine upper respiratory tract. Part 1: observations during high-speed treadmill endoscopy of 600 thoroughbred racehorses. Equine Vet J. (2006) 38:393–9. 10.2746/042516406778400583 16986598

[5] Ducharme NG. Pharynx. In: Auer JA Stick JA editors. Equine Surgery, 4th ed. Philadelphia, PA: Sounders Elsevier (2012). p. 561–90.

[6] Davison JA Lumsden JM Boston RC Ahern BJ. Overground endoscopy in 311 thoroughbred racehorses: findings and correlation to resting laryngeal function. Aust Vet J. (2017) 95:338–42. 10.1111/avj.12620 28845565

[7] Stick JA Peloso JG Morehead JP. Endoscopic assessment of airway function as a predictor of racing performance in Thoroughbred yearlings: 427 cases (1997–2000). J Am Vet Med Assoc. (2001) 219:962–6. 10.2460/javma.2001.219.962 11601794

[8] Hackett ES Leise BS Exercising upper respiratory video endoscopic findings of 50 competition draught horses with abnormal respiratory noise and/or poor performance. Equine Vet J. (2019) 51:370–74. 10.1111/evj.13026 30267613

[9] Cheetham J Dorsal displacement of the soft palate: pathophysiology and new diagnostic techniques. In: Hawkins J editor. Advances in Equine Upper Respiratory Surgery. 1st ed. Indianapolis, IN: John Wiley & Sons (2015). p. 89–95.

[10] Ducharme NG Hackett RP Woodie JB The investigation into the role of the thyrohyoid muscles in the pathogenesis of dorsal displacement of the soft palate. Equine Vet J. (2003) 35:258–63. 10.2746/042516403776148200 12755428

[11] Woodie JB Ducharme NG Kanter P Hackett RP Erb NH. Surgical advancement of the larynx (laryngeal tie-forward) as a treatment for the dorsal displacement of the soft palate in horses: a prospective study 2001-2004. Equine Vet J. (2005) 37:418–23. 10.2746/042516405774480076 16163943

[12] Cheetham J Pigott JH Thorson LM Mohammed HO Ducharme NG. Racing performance following the laryngeal tie-forward procedure: a case-controlled study. Equine Vet J. (2008) 40:501–11. 10.2746/042516408X313616 18490235

[13] Holcombe SJ Derksen FJ Stick JA Bilateral nerve blockade of the pharyngeal branch of the vagus nerve produces persistent soft palate dysfunction in horses. Am J Vet Res. (1998) 59:504–8. 9563638

[14] Cheetham J Pigott JH Hermanson JW Campoy L Soderholm LV Thorson LM. Role of the hypoglossal nerve in equine nasopharyngeal stability. J Appl Physiol. (2009) 107:471–7. 10.1152/japplphysiol.91177.2008 19498094

[15] Gille D Lavoie JP Review of seven cases of ulcers of the soft palate. Equine Pract. (1996) 18:9–13.

[16] Blythe LL Cardinet GH Meagher DM. Palatal myositis in horses with dorsal displacement of the soft palate. J Am Vet Med Assoc. (1983) 183:781–5. 6629986

[17] Holcombe SJ Derksen FJ Stick JA Robinson NE. Effects of bilateral hypoglossal and glossopharyngeal nerve blocks on epiglottic and soft palate position in exercising horses. Am J Vet Res. (1997) 58:1022–6. 9285009

[18] Duncan DW Retrospective study of 50 Thoroughbred racehorses subjected to radical myectomy surgery for treatment of displacement of the soft palate. Proc Am Assoc Equine Pract. (1997) 43:237–8.

[19] Llewellyn HR Petrowitz AB Sternothyroideus myotomy for the treatment of dorsal displacement of the soft palate. Proc Am Assoc Equine Pract. (1997) 43:239–43.

[20] Harrison IW Raker CW. Sternothyrohyoideus myectomy in horses: 17 cases (1984-1985). J Am Vet Med Assoc. (1988) 193:1299–302. 3204058

[21] Anderson JD Tulleners EP Johnston JK Reeves MJ. Sternothyrohyoideus myectomy or staphylectomy for treatment of intermittent dorsal displacement of the soft palate in racehorses: 209 cases (1986-1991). J Am Vet Med Assoc. (1995) 206:1909–12. 7790306

[22] Ahern TJ Oral palatopharyngoplasty. J Equine Vet Sci. (1993) 13:185–8. 10.1016/S0737-0806(06)81000-2

[23] Ducharme NG Update on the laryngeal tie-forward operation. In: Proceedings of the Equine Upper Airway Symposium. Lexington, KY Rood and Riddle Foundation (2013). p. 1–3.

[24] Ahern BJ Parente EJ. Surgical complications of the equine upper respiratory tract. Vet Clin North Am Equine Pract. (2008) 24:465–84. 10.1016/j.cveq.2008.10.004 19203696

[25] Santos MP Gutierrez-Nibeyro SD Horn GP Hicke JD Stewart MC Schaeffer DJ. *In vitro* mechanical evaluation of equine laryngeal tie-forward constructs prepared with different suture materials and placement patterns. Am J Vet Res. (2015) 76:373–83. 10.2460/ajvr.76.4.373 25815579

[26] Santos MP Gutierrez-Nibeyro SD Horn GP Wagoner Johnson AJ Stewart MC Schaeffer DJ. Mechanical properties of various suture materials and placement patterns tested with surrogate *in vitro* model constructs simulating laryngeal advancement tie-forward procedures in horses. Am J Vet Res. (2014) 75:500–6. 10.2460/ajvr.75.5.500 24762024

[27] Rossignol F Ouachee E Boening KJ. A modified laryngeal Tie-forward procedure using metallic implants for the treatment of dorsal displacement of the soft palate. Vet Surg. (2012) 41:685–8. 10.1111/j.1532-950X.2012.01001.x 22823753

[28] Ahern BJ Boston RC Parente EJ. *In vitro* mechanical testing of an Alternate Laryngoplasty System (ALPS) for Horses. Vet Surg. (2012) 41:918–23. 10.1111/j.1532-950X.2012.01061.x 23198920

[29] Secor EJ Gutierrez-Nibeyro S Horn GP. Biomechanical evaluation of modified laryngoplasty by use of a toggle technique for stabilization of arytenoid cartilage in specimens obtained from equine cadavers. Am J Vet Res. (2018) 79:226–32. 10.2460/ajvr.79.2.226 29359970

[30] Chan RW Titze IR. Effect of postmortem changes and freezing on the viscoelastic properties of vocal fold tissues. Ann Biomed Eng. (2003) 31:482–91. 10.1114/1.1561287 12723689

[31] Changoor A Fereydoonzad L Yaroshinsky A Bushmann MD. Effects of refrigeration and freezing on the electromechanical and biomechanical properties of articular cartilage. J Biomech Eng. (2010) 132:1–6. 10.1115/1.4000991 20887036

[32] Szarko M Muldrew K Bertram JEA. Freeze-thaw treatment effects on the dynamic mechanical properties of articular cartilage. BMC Musculoskelet Disord. (2010) 11:1–8. 10.1186/1471-2474-11-231 20932309 PMC2958988

[33] Wuest DM Meyer DC Favre P Gerber C Mechanical and handling properties of braided polyblend polyethylene sutures in comparison to braided polyester and monofilament polydioxanone sutures. Arthroscopy. (2006) 22:1146–53. 10.1016/j.arthro.2006.06.013 17084288

